# Methylene Blue-Aided In Vivo Staining of Central Airways during Flexible Bronchoscopy

**DOI:** 10.1100/2012/625867

**Published:** 2012-08-01

**Authors:** Sabine Zirlik, Kai M. Hildner, Markus F. Neurath, Florian S. Fuchs

**Affiliations:** Department of Medicine, University of Erlangen-Nuremberg, Ulmenweg 18, 91054 Erlangen, Germany

## Abstract

*Background*. The early diagnosis of malignant and premalignant changes of the bronchial mucosa remains a major challenge during bronchoscopy. Intravital staining techniques are not new. Previous small case series suggested that analysis of the bronchial mucosal surface using chromoendoscopy allows a prediction between neoplastic and nonneoplastic lesions. *Objectives*. The aim of the present study was to evaluate chromobronchoscopy as a method to identify malignant and premalignant lesions in the central airways in a prospective manner. *Methods*. In 26 patients we performed chromoendoscopy with 0.1% methylene blue during ongoing flexible white light bronchoscopy. Circumscribed lesions in central airways were further analyzed by biopsies and histopathologic examination. *Results*. In the majority of cases neither flat nor polypoid lesions in the central airways were stained by methylene blue. In particular, exophytic growth of lung cancer did not show any specific pattern in chromobronchoscopy. However, a specific dye staining was detected in one case where exophytic growth of metastatic colorectal cancer was present in the right upper lobe. In two other cases, a circumscribed staining was noted in unsuspicious mucosa. But histology revealed inflammation only. *Conclusions*. In contrast to previous studies, the present findings clearly indicate that chromobronchoscopy is not useful for early detection of malignant or premalignant lesions of the central airways.

## 1. Introduction

The diagnostic value of flexible bronchoscopy for various pulmonary diseases is well established. This method is especially important in detecting malignant lesions including their precursors in the central airways. Lung cancer has still a very poor prognosis compared to other types of cancer. Many patients get diagnosed in an advanced disease stages for lack of symptoms [1–3]. There is a high need for the development of new endoscopic techniques, which provide a reliable and easy detection of premalignant lesions and early stages of lung cancer in the central airways.

There is no doubt that patients with distinct mucosal neoplasia could develop an invasive carcinoma as it could have been shown by a rate of 40–83% [4–6]. To improve detection of these premalignant lesions, autofluorescence bronchoscopy (AFB) and narrow band imaging [7–10] were extensively evaluated in the last years. A relevant number of false negative results especially in AFB and additional acquisition costs for the systems are the major limitations for these techniques.

In gastroenterology staining with methylene blue (MB) during endoscopy is a well-established method. It offers a detailed analysis of the colonic mucosal surface and pit pattern architecture. Chromoendoscopy in the gut allows a prediction between neoplastic and nonneoplastic lesions with high specificity [11]. More than 20 years ago two studies were published regarding bronchial mucosa [12, 13]. Their data proofed that a distinction was possible between normal and pathological mucosa by the use of MB during white light bronchoscopy (WLB). In both studies the majority of patients showed exophytic tumour growth. Histology mostly revealed squamous cell carcinoma. Despite these encouraging early reports, staining during WLB (chromobronchoscopy) has not been added to the diagnostic panel of pulmonary endoscopy up to now. This is worth mentioning because chromobronchoscopy would be a cheap and easy technique in support of WLB for detection of early-stage central lung cancer. The aim of this study was to evaluate the impact of chromobronchoscopy with MB in unselected patients with various indications for WLB.

## 2. Patients and Methods

Flexible bronchoscopy was performed in 26 consecutively recruited patients (10 women, 16 men; age 47 to 82 years). Bronchoscopy was done under intravenous sedation (midazolam 3 to 7 mg, additionally pethidine 50 to 100 mg or etomidate 4 to 12 mg) with a video chip endoscope (Olympus BF 1T180 or BF Q180, Olympus, Tokyo, Japan). The patients were monitored via ECG, pulse oximetry, and intermittent noninvasive measurement of blood pressure. Every person received at least 2 L O_2_/min via nasal O_2_ tube. Topical anaesthesia was carried out with lidocaine solution limited to 4.5 mg/kg body weight.

First WLB was performed as usual. All macroscopic findings including signs of general inflammation (redness, hypervascularisation) or malignancy (exophytic tumour growth, irregularity of the bronchial mucosa, nodular or polypoid lesions, thickening of a carina, and atypical vascularisation) were documented. Then chromobronchoscopy was performed. Secretion was extensively aspirated from the bronchial system. Then 5 mL acetylcysteine 10% was applied through the bronchoscope. After 1 minute airways were washed with 10 mL saline solution 0.9% followed by another aspiration of remaining fluids. Thereupon 10 mL MB 0.1% was applied with a spray catheter. As we saw a marked breathing-dependent endogenous distribution of the dye in the first cases, we subsequently applied it into the mainstem bronchi with a sufficient result. After one minute of exposure, the stained area was washed again with 10 mL saline solution 0.9%. After removing all fluids again the whole airways were inspected once more. According to former findings in AFB, we defined a circumscribed and remarkable uptake of the dye to be possibly malignant. Biopsies were taken from such areas as well as from all other possibly malignant lesions in WLB.

The study was approved by the ethical committee of the University hospital Erlangen. All patients gave written informed consent.

## 3. Results

All patients underwent WLB and chromobronchoscopy according to the study protocol. No side effects of the staining procedure occurred during bronchoscopy or during a followup of 24 hours in all patients. In 11 cases either exophytic tumour growth or carcinomatosis of the mucosa was found in WLB. Twelve patients showed only signs of mucosal atrophy in WLB. Further pathologic findings were esophagotracheal fistula due to esophagus cancer, stenosis of the trachea and the left mainstem bronchus by compression in a patient with sarcoidosis, and tracheobronchial papillomatosis. Underlying diseases are listed in Table 1.

**Figure 1:**
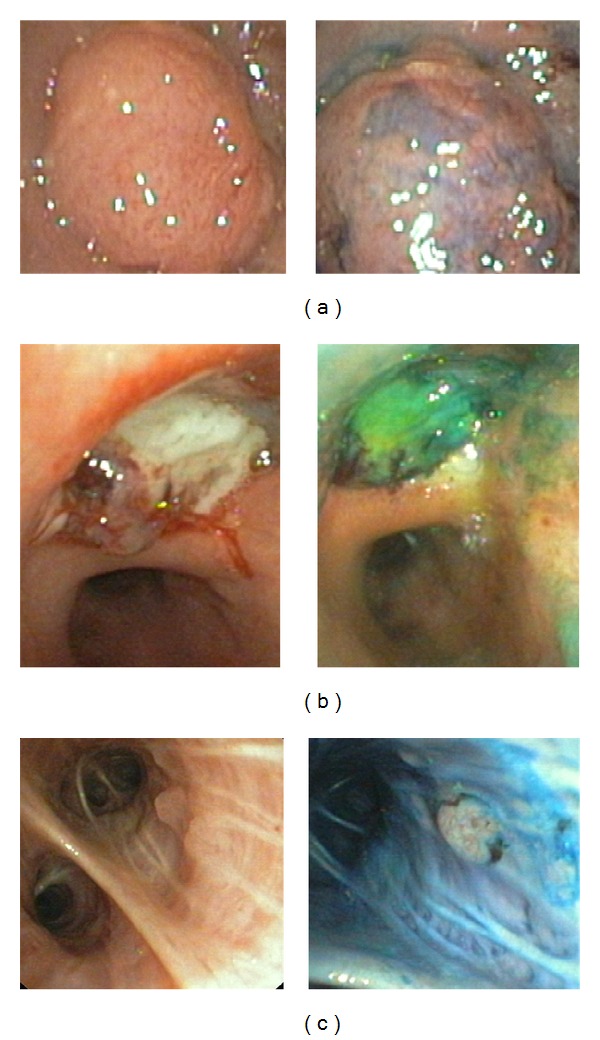
(a) Occluding tumour growth in the mainstem left bronchus (squamous cell cancer of the lung) in white light bronchoscopy (left) and chromobronchoscopy after staining with methylene blue (right). The tumour shows only small areas with a weak blue staining. The main part of the tumour remained unstained. (b) Occluding tumour growth in the right upper lobe (metastasis of colorectal cancer) in white light bronchoscopy (left) and chromobronchoscopy after staining with methylene blue (right). The tumour is stained bright blue to green whereas the noninfiltrated mucosa remained largely unstained. (c) Small exophytic tumour growth in the right lower lobe bronchus (adenocarcinoma of the lung) in white light bronchoscopy (left) and chromobronchoscopy after staining with methylene blue (right). In contrast to the surrounding mucosa, the malignant tissue remained unstained.

In all patients a weak and nonspecific staining with MB of the whole bronchial mucosa could be found. In particular, exophytic growth of lung cancer (small cell and nonsmall cell) did not show any specific pattern in chromobronchoscopy (Figure 1(a)). Areas with malignant infiltration of the mucosa remained also unstained in lung cancer and in one case with metastasic renal cell cancer. So, chromobronchoscopy could not provide an improved identification of the extent of mucosal tumour growth in such cases. All obviously malignant lesions detected during WLB cancer could be confirmed in histopathological examination. Only in one case tumour tissue showed a considerable MB uptake (Figure 1(b)). It was diagnosed as an exophytic metastasis of known colorectal cancer in histology. In another case tumour tissue remained unstained whereas normal mucosa showed a homogenous bright blue staining (Figure 1(c)).

**Figure 2:**
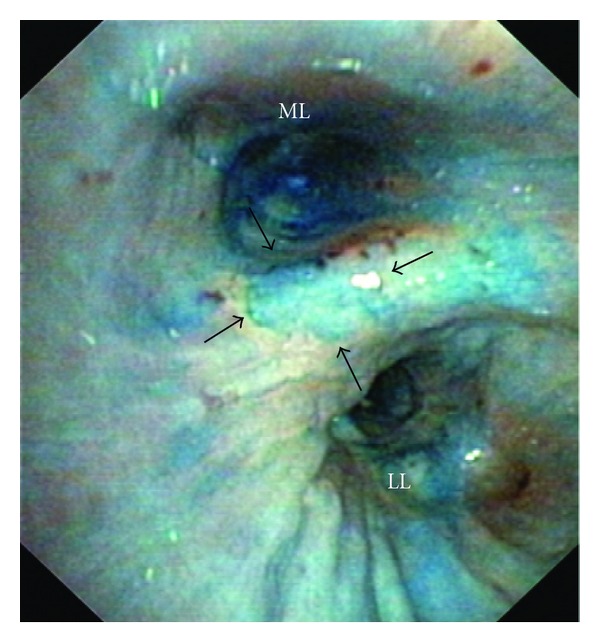
Circumscribed staining (arrows) in chromobronchoscopy; ML: middle lobe, LL: lower lobe; histology showed unspecific inflammation.

In those patients without signs of malignancy in WLB, there was a circumscribed mucosal staining in two cases. But histological examination only showed signs of inflammation (Figure 2). In tracheobronchial papillomatosis also no staining could be detected. There were no signs of malignant transformation into squamous cell carcinoma in histology of the removed tissue.

## 4. Discussion

In this study we evaluated the intravital staining technique with MB (chromobronchoscopy) for detection of malignant and premalignant lesions in unselected patients. In contrast to former studies, we could not show any diagnostic benefit by this method which has become standard in gastrointestinal endoscopy.

More than 20 years ago, Ovchinnikov et al. and Varoli et al. [12, 13] described successful usage of MB for chromobronchoscopy in patients with exophytic tumour growth. But this technique was never applied routinely during WLB. One cause could be that the results were not reproducible. A slow and deficient knowledge exchange between gastrointestinal and pulmonary endoscopy could be another cause why chromobronchoscopy was not developed until now. Confocal laser endomicroscopy (CLE) is a good example for this phenomenon. While this method has almost become a standard technique in gastroenterology [14], its usage is widely experimental in the lung without a clearly defined field of application. Only few reports exist about the use of pulmonary CLE [15–17]. Recently we could show that addition of fluorescein during CLE does not provide any advantage in imaging of the central airways [18].

In nearly all patients there was only a nonspecific background mucosal staining. In two cases there was a staining pattern, which was suspicious according to our definition. But histology only showed inflammation. Remarkably the only lesion which showed a significant staining was an exophytic growing metastasis of colorectal cancer. Such a different behaviour of adenocarcinoma of the lung and the gastrointestinal tract is already known from other aspects, for example, sensitiveness against specific cytostatic drugs or preferred locations of metastasis. So our findings may at least be helpful to differentiate exophytic growth of NSCLC or metastasis of colorectal cancer during bronchoscopy, although this can be done easily by immunohistochemistry.

We used MB for staining of central airways as it is a well-known substance for chromoendoscopy in gastroenterology. Furthermore MB appears to be an appropriate dye for chromobronchoscopy. There are sufficient experiences on topical pulmonary application. The restricted value of MB dye in bronchoscopy may be related to the lower and partially missing number of goblet cells compared to the intestinal tract. Maybe other dyes are more useful for staining during chromobronchoscopy. Recently the use of cresyl violet was reported to be an interesting alternative dye for chromoendoscopy [19] as endomicroscopy can be performed simultaneously with this substance. However, safety of topical applied cresyl violet in the lung is not known yet. This should be topic of further studies. Chromobronchoscopy might be a helpful method when performed with another dye. No high technical standard is needed to install it in routine clinical practice.

To conclude chromobronchoscopy with MB is not helpful for early detection of malignant or premalignant lesions of the bronchial system. These findings explain why it has not become a standard method in pulmonary endoscopy until now although former studies showed positive results. However, our findings should encourage further research on chromobronchoscopy. There should also be an intensified knowledge transfer between gastrointestinal and pulmonary endoscopy.

## Figures and Tables

**Table 1:** Underlying diseases which were indications for bronchoscopy.

*n*	Underlying disease
6	Adenocarcinoma of the lung
4	Squamous cell cancer of the lung
2	Small cell lung cancer
2	Bronchoalveolar carcinoma
1	Metastasis of renal cancer
1	Metastasis of colorectal cancer
1	Papillomatosis of the trachea
2	Esophageal cancer
2	Carcinoma of unknown primary
1	Sarcoidosis
2	Followup after lung cancer surgery
2	Suspicious findings in CT scan
